# In situ investigation of phase transformations in Ti-6Al-4V under additive manufacturing conditions combining laser melting and high-speed micro-X-ray diffraction

**DOI:** 10.1038/s41598-017-16760-0

**Published:** 2017-11-27

**Authors:** C. Kenel, D. Grolimund, X. Li, E. Panepucci, V. A. Samson, D. Ferreira Sanchez, F. Marone, C. Leinenbach

**Affiliations:** 1 Northwestern University - Department of Materials Science and Engineering, 2200 Campus Drive, Evanston, IL 60208 USA; 2 Empa - Swiss Federal Laboratories for Materials Science and Technology, Überlandstrasse 129, 8600 Dübendorf, Switzerland; 3 Paul Scherrer Institut, Swiss Light Source, 5232 Villigen, PSI Switzerland

## Abstract

We present combined *in situ* X-ray diffraction and high-speed imaging to monitor the phase evolution upon cyclic rapid laser heating and cooling mimicking the direct energy deposition of Ti-6Al-4V in real time. Additive manufacturing of the industrially relevant alloy Ti-6Al-4V is known to create a multitude of phases and microstructures depending on processing technology and parameters. Current setups are limited by an averaged measurement through the solid and liquid parts. In this work the combination of a micro-focused intense X-ray beam, a fast detector and unidirectional cooling provide the spatial and temporal resolution to separate contributions from solid and liquid phases in limited volumes. Upon rapid heating and cooling, the β ↔ α′ phase transformation is observed repeatedly. At room temperature, single phase α′ is observed. Secondary β-formation upon formation of α′ is attributed to V partitioning to the β-phase leading to temporary stabilization. Lattice strains in the α′-phase are found to be sensitive to the α′ → β phase transformation. Based on lattice strain of the β-phase, the martensite start temperature is estimated at 923 K in these experiments. Off-axis high speed imaging confirms a technically relevant solidification front velocity and cooling rate of 10.3 mm/s and 4500 K/s, respectively.

## Introduction

Additive manufacturing (AM) of metals is increasingly applied to produce complex parts in aerospace, defence, medical and automotive industries. The beam-based AM technologies for metals can be distinguished into powder-bed fusion (PBF) and directed energy deposition (DED) technologies where material is fed into the melt pool^1,2^. While PBF is restricted to a flat building plane due to its gravity-compacted powder bed, DED allows for processing comparable to CNC machining with freedom of movement in multiple axes. In a typical DED process, material is fed as powder or wire into the melt pool heated by a laser, arc or electron beam. The complete processing head and the work piece are then moved along the tool path to create the geometrically complex part. Compared to PBF, higher build rates are achieved and larger components up to several meters in size can be produced. The layer-wise fashion of DED imposes a cyclic thermal history, with reported cooling rates between 10^2^–10^5^ K s^−1^, affecting phase evolution, microstructure formation and ultimately the achievable properties^3^.

A material with a high technical relevance in the context of AM is the (α + β) dual phase titanium alloy Ti-6Al-4V^1,4^. Depending on the thermo-mechanical history, the microstructure of Ti-6Al-4V can be complex with features spanning different length scales, influencing the mechanical properties^5^. In comparison with conventional manufacturing technologies like casting or forging, the microstructures of additive manufactured Ti-6Al-4V can be particularly complex with a variety of different phases (hcp α, bcc β, hexagonal α’ martensite, orthorhombic α” martensite, Ti_3_Al), different morphologies (e.g. elongated α grains, regions of massive α containing nano-sized β) and often anisotropic mechanical properties, depending on the AM technology and the processing parameters^6–15^. However, there is still a lack of understanding of the phase transformation sequence – not only in Ti-6Al-4V, but also in many other technically relevant alloys – involved in the cyclic rapid heating and cooling of a small material volume during AM.

*In situ* synchrotron X-ray diffraction and radiographic imaging is increasingly applied to reveal the non-equilibrium nature and dynamics of AM processing^16–21^. The most advanced setup reported so far combines *in situ* high-speed synchrotron X-ray radiography and diffraction to reveal the dynamics of melt pool formation in PBF of Ti-6Al-4V^19^. Unfortunately, the wide beam used for imaging results in diffraction over a wide area. This complicates specific temperature-phase correlation due to diffraction data being collected over a wide area and thus a wide temperature range. Additionally, deposited material is subjected to cyclic thermal treatments upon deposition of subsequent layers. This aspect is not covered by setups reported so far.

In this work, we report *in situ* probing of the phase evolution in cyclically rapidly laser heated and cooled Ti-6Al-4V mimicking real AM DED conditions by combined *in situ* synchrotron micro-focused X-ray diffraction and high-speed imaging at the Swiss Light Source (SLS). It is demonstrated that the high spatial and temporal resolution allows observation of the cyclic phase evolution upon multiple thermal cycles and provides information about non-equilibrium transformation dynamics and structural information on the formed phases. It is also demonstrated that the achieved cooling rate and solidification speeds are directly comparable to real processing conditions. The experimental setup and data analysis procedures developed in this work will provide high-value additional data on the fundamental understanding of the AM process not available from currently deployed setups.

## Results and Discussion

### *In situ* high speed synchrotron micro X-ray diffraction and imaging

**Figure 1:**
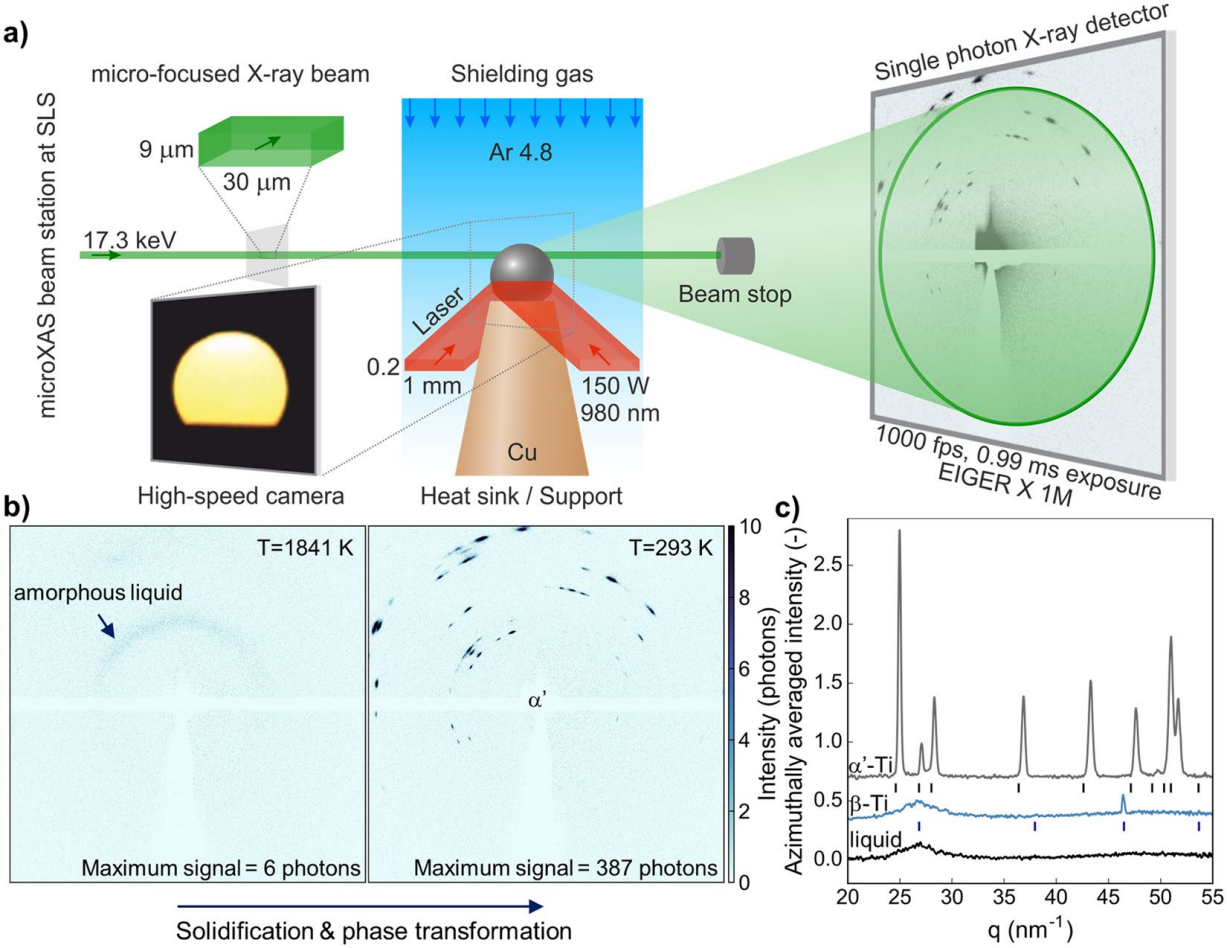
Laser melting combined with *in situ* high-speed micro X-ray diffraction and imaging. The specimen, supported on a copper tip acting as heat sink, is rapidly heated using two inclined diode lasers while passing through a micro-focused X-ray beam. Diffracted intensities are recorded using an EIGER × 1 M single photon counting detector. Off-axis high-speed imaging provides surface temperature distribution and laser positioning control (**a**). Time series of recorded 2D diffractograms provide information on phase evolution from liquid to the solid state (**b**). Data shown is from the undercooled liquid (T = 1841 K) prior to β nucleation and at room temperature. All data are background subtracted and reduced to 1D diffractograms by azimuthal integration. Diffractograms for liquid (bottom), β-Ti (middle) and α′-Ti (top) are shown with the ideal peak positions for Ti (**c**). Shifts are caused by reduced lattice parameters by V addition.

The experimental setup at the microXAS beamline at the Swiss Light Source at the Paul Scherrer Institut (Switzerland) combines rapid laser heating and subsequent rapid solidification of the Ti-6Al-4V specimen, *in situ* transmission X-ray diffraction using a micro-focused beam and high-speed imaging (Fig. 1a). Two inclined diode lasers focused onto the specimen provide the necessary power input for melting and subsequent heat treatments. All laser heating and cooling cycles are performed on the same specimen in rapid succession with 10 s measurement time per cycle. Ar shielding gas is provided by a laminar flow nozzle in a radius of 15 mm around the specimen. By this approach the complete setup is window-less to minimize intensity losses and maximize temporal resolution. The specimen is supported on a Cu tip acting as a heat sink that rapidly cools the small amount of hot material after the lasers are shut-off. During the complete heating-cooling cycle, a 17.3 keV micro-focused X-ray beam is passed through the specimen and diffracted intensities are recorded by the EIGER × 1 M single-photon counting detector with 1030 × 1065 pixels operating at a frame rate of 1 kHz and 0.99 ms exposure per frame. Recorded raw frames reveal scattered signals from amorphous and crystalline phases combined with features from beam optics, the specimen holder, the beam stop, defective pixels and the module border that are removed by a background correction (Fig. 1b and Supplementary Figure S1). The unit of the recorded intensities is single photons as the EIGER detector directly counts photons exceeding a threshold energy (8.7 keV in these experiments) to supress low-energy X-rays. The low background of typically 0 to 2 photons per pixel after 0.99 ms exposure time in combination with the high photon flux of the microXAS beam station (2 × 10^12^ ph/s/400 mA) enables the high temporal resolution in the performed experiments. The weak diffuse scattering signal from the amorphous liquid phase with a maximum signal intensity of 6 photons as well as the comparably strong signal from solid (crystalline) α′-Ti with a maximum of 387 photons lead to 1D diffractograms with good signal-to-noise ratio after background subtraction and azimuthal integration and averaging (Fig. 1c). Peak shifts to higher values of the momentum transfer *q* compared to the diffractogram of pure Ti are caused by lattice contraction upon alloying with V^22^.

High-speed camera imaging with 500 Hz frame rate is performed 45° off-axis with respect to the X-ray beam direction. Based on the recorded intensity, temperature is calculated based on a radiation model and camera calibration^23^. The temperature distribution on the surface as well its evolution upon cooling provides information about thermal gradients and obtained cooling rates. Uncertainties in obtained diffractograms due to thermal gradients are estimated to be ±15 K horizontally along the x-ray beam and ±4 K vertically, including thermal expansion of the specimen (see Supplementary). Additionally, the camera provides remote control over laser positioning and simplifies alignment procedures.

### Phase evolution upon rapid cyclic heating and cooling

**Figure 2:**
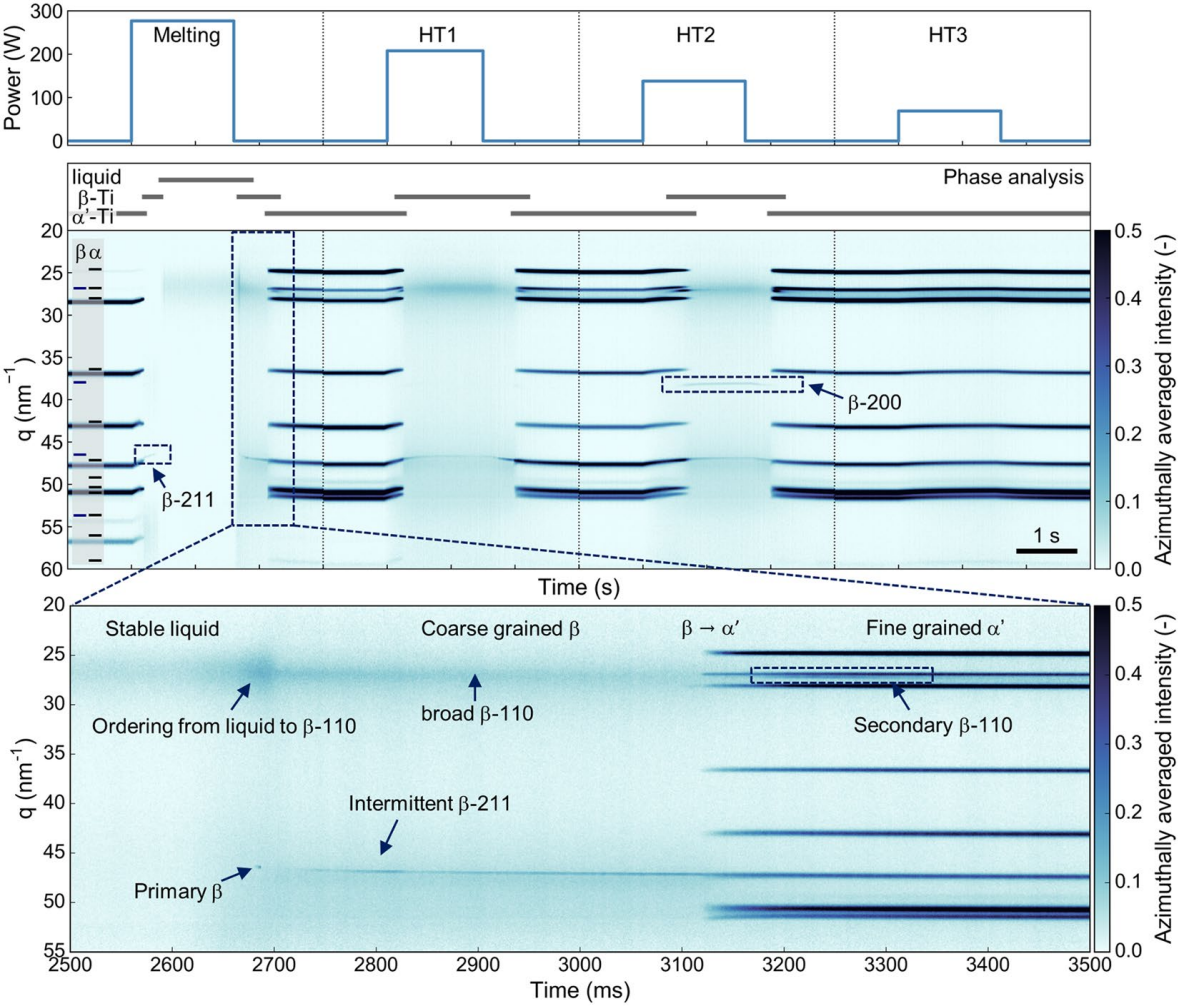
Phase evolution upon cyclic laser heating The specimen is cyclically heated and cooled using decreasing laser powers. After initial melting and rapid solidification, the material is subjected to three heat treatments with decreasing heat input. The resulting phase evolution is shown as a scattering vector q vs. time stitched film plot of 4 s segments by stacking the 1D diffractograms for each time point. Upon heating α′-Ti transforms to β-Ti which fully melts in the first cycle but remains stable in HT1 and HT2. In HT3 the maximum temperature does not exceed the β-transus and no β-Ti is observed. A magnified section shows formation of a stable liquid, rapid formation of a β-grain followed by full solidification and β → α′ transformation.

Materials subjected to layer-by-layer beam based AM are subjected to cyclic heat treatments. First, upon deposition melting is initiated; later deposition of subsequent layers thermally affects the underlying material which acts as the heat sink. Consequently, bulk AM material has a complex thermal history that exceeds a pure rapid solidification step as covered by state-of-the art setups. In this work, the specimen is subjected to a cyclic laser heating and subsequent cooling, mimicking the layer-wise processing in AM (Fig. 2, top). Laterally stacked 1D diffractograms covering 4 s in every cycle form the presented 2D film plot with a total length of 16 s to reveal the phase evolution throughout the cyclic thermal treatments. Initially the specimen consists of α-Ti or martensitic α′-Ti phase based on the obtained diffraction pattern. As both phases are hexagonal and have similar lattice parameters, they cannot be distinguished by diffraction. It is known that quenched Ti-6Al-4V displays a martensitic transformation, therefore it is assumed that the spheres will consist of α′ martensite upon rapid cooling^22^. In the first cycle (melting), the specimen rapidly transforms to β-Ti as observed by a weak β-211 peak and later melts as revealed by the broad diffraction intensity between 25 and 30 nm^−1^ (Fig. 2, centre). This sequence is reversed upon cooling. First, increased diffraction intensity and a slight shift in *q* reveals ordering from the amorphous liquid to the β-phase (Fig. 2, bottom). This primary β coexists with the liquid upon solidification and is observed intermittently before stabilizing around 2850 ms in the first cycle. The β-phase appears to be coarse grained based on the low recorded intensity of only a few grains fulfilling the diffraction condition. After 3130 ms the full range of α′-phase peaks appears revealing a fine-grained nature. With the appearance of α′, a secondary increase in β-110 is observed.

In the second cycle with reduced laser intensity (HT1) the alloy fully transforms to β as indicated by the continuous presence of the β-211 diffraction peak. Upon cooling, the alloy again undergoes the β → α′ transformation. In the third cycle (HT2), the specimen temperature still exceeds the β-transus and the α′ → β transformation is observed. Compared to the melting step and HT1, the phase transformation appears to become sluggish as α′ and β coexist for about 500 ms until the transformation is complete. The presence of β-200, which was not observed after melting and only weakly in HT1, indicates either grain rotation or grain refinement in the β-phase upon high temperature heat treatment. In the fourth cycle (HT3), the temperature does not exceed the β-transus and only thermal expansion of the α′-phase is observed.

**Figure 3:**
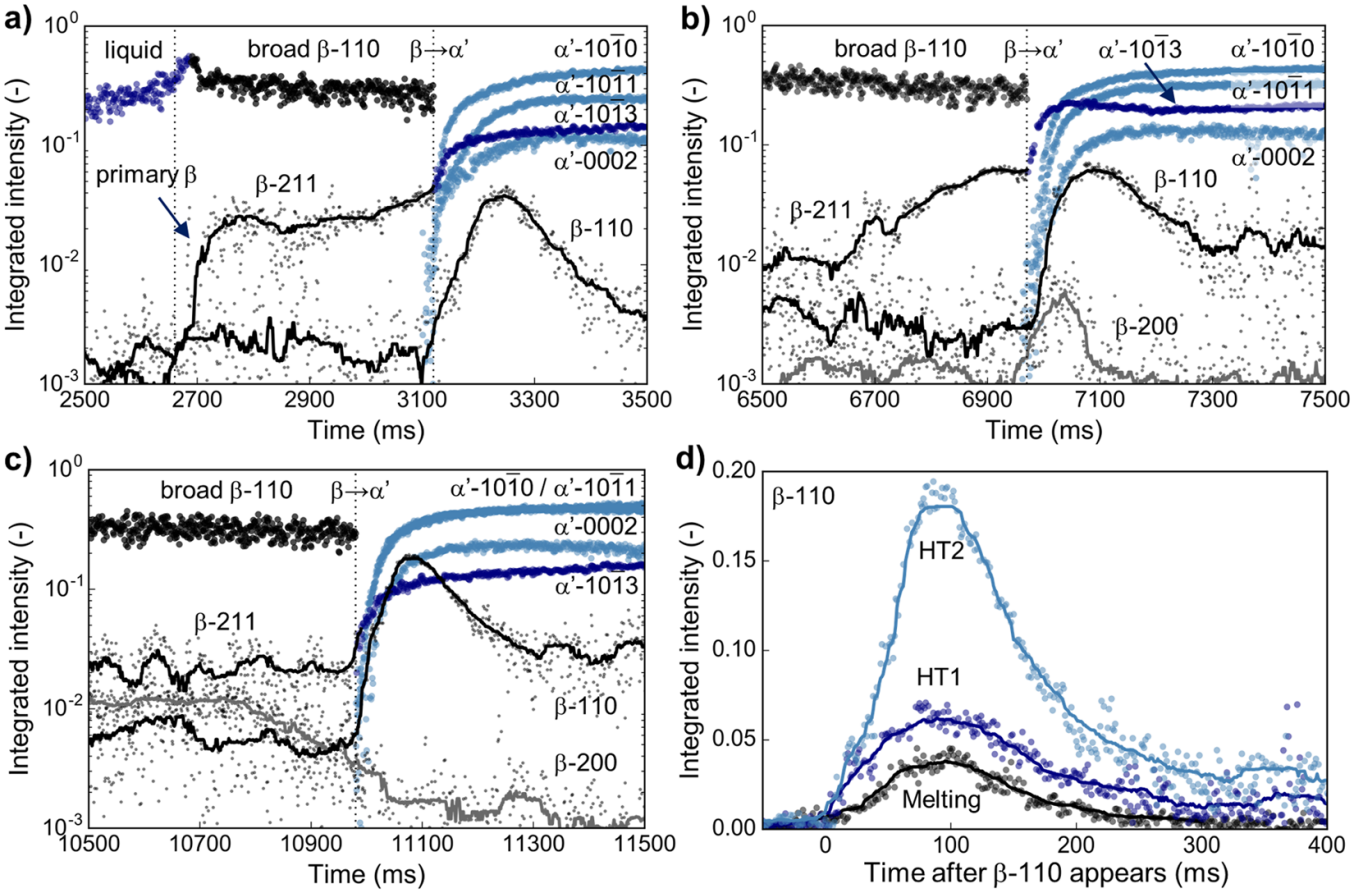
Peak evolution upon rapid cooling The evolution of the integrated peak intensities is obtained by fitting the 1D diffractograms for every time point. For the melting cycle, β-Ti is obsereved after stable melt formation. Upon fast β → α′ transformation, the intensity of β-110 sharply increases before fading away (**a**). In HT1 weak diffraction spots from β-Ti are observed at high temperature. Upon β → α′ again β-110 and β-200 temporarily increase (**b**). For HT2 β is clearly observed throughout the high temperature period with a sharp rise in β-110 upon β → α′ (**c**). Comparison of the β-110 intensity shows increased diffraction intensity from melting over HT1 to HT2 with step-wise decreased energy input and temperature (**d**). Weak diffraction intensities from β-Ti are shown with a rolling median as visual guide.

A detailed analysis of the integrated peak intensities and their evolution in time upon melting, HT1 and HT2 is shown in Fig. 3. Prior to solidification in the melting cycle, all crystalline β-peaks are at the background noise level around 2·10^−3^ (Fig. 3a). Peaks from the α′-phase are ommited and weak β-peaks are shown with a rolling median for clarity. Upon soldification, the intensity in the broad amorphous liquid peak increases and reaches a maximum at 2700 ms. After this point this peak is identified as a broad, diffuse β-110 diffraction intensity containing contributions from diffuse scattering close to a Bragg reflection as well as intensity from the diffuse signal of a densely packed body-centered cubic structure^24^. Simultaneously, β-211 sharply rises reaching a plateau around 2·10^−2^ (Note the logarithmic scale of the intensity). At 3100 ms all α′-peaks sharply rise by two orders of magnitude and stabilize by 3300 ms. Only four α′-peaks are shown for clarity and α′-10 $\bar{1}$3 is shown in dark blue as it overlaps with β-211. While the broad diffuse β-110 and the weak β-211 cannot be distinguished anymore from the strong overlapping α′-peaks, a distinct β-110 peak is observed after the β → α′ transformation started. Due to its observation after the broad and/or weak β-peaks disappear and α′ appears this is termed secondary β.

Upon cooling after HT1, β-211 continuously rises after 6650 ms. The time is adapted from Fig. 2 to distinguish the different cycles. Upon β → α′, β-200 reaches a maximum around 6·10^−3^ and disappears again within 150 ms. Also starting with the transformation, the distinct secondary β-110 peak is observed again. Upon HT2, β-211 slightly fluctuates around a plateau value while β-200 decreases upon cooling (Fig. 3c). The secondary β-110 peak is observed again reaching intensities larger than α′-0002 and α′-10 $\bar{1}$3. After β → α′ in HT1, α′-10 $\bar{1}$1 and αα′-10 $\overline{1}$3 reveal increased intensities compared to the melting cycle, while other peaks reach similar values.

**Figure 4:**
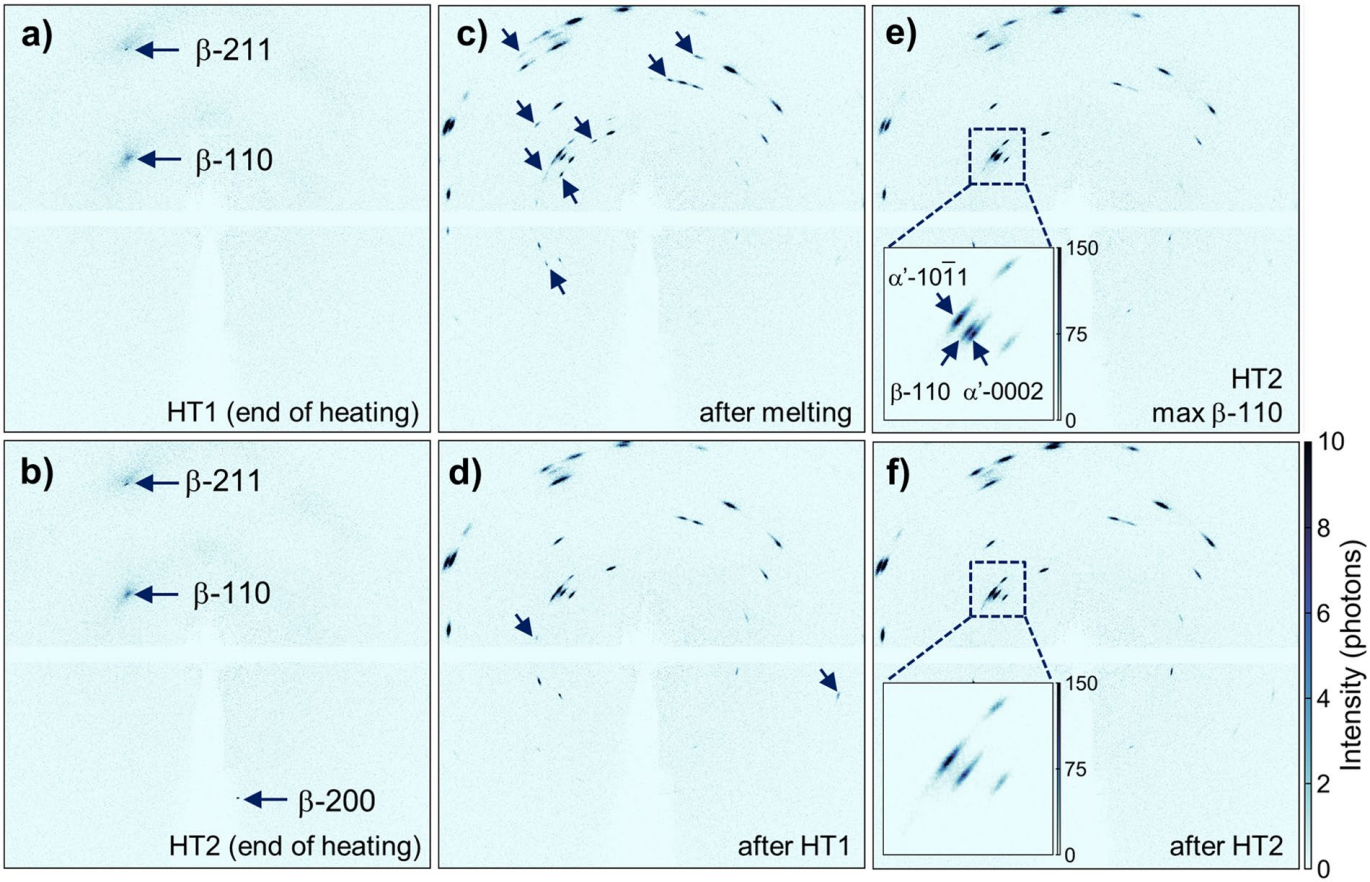
Evolution of 2D diffractograms upon thermal cycling 2D diffractograms as recorded by the EIGER module are used to evaluate the structural evolution upon thermal cycling. At the end of the heating stage in HT1 (T = 1778 K) coarse β is present (**a**). After HT2 (T = 1314 K), the β-200 reflection is observed (**b**). Hexagonal α′ formed after rapid solidification (**c**) shows removal of weak reflections close to intense ones (**d**) thus continously reducing the number of sub-grains (**e**,**f**). The β and α′ phases show a clear orientation relationship (inset **e**) before β is fully transformed (inset f). Selected disappearing reflections are marked with arrows.

After HT2 the α′-10 $\overline{1}$1 again increases compared to the melting step and HT1. For a perfect XRD powder pattern this peak would be expected to be the strongest one for the hexagonal phase. Its increase with every cycle coincides with removal of weak reflection from the 2D patterns and increased intensity of remaining, favourable grain orientations (Fig. 4). Figure 3d shows a comparison of the observed secondary β-110 peaks upon melting, HT1 and HT2. The maximum intensity shows a clear dependency on the applied laser power. With decreasing laser power, thus reduced maximum temperature and prolonged time in the β-phase, β-110 increases. Independent of the reduced laser power in the different cycles, the maximum intensity is observed around 100 ms after the peak is clearly observed before fading away after 300 ms. This effect is explained by work of Kabra *et al*. performing *in situ* neutron diffraction on structurally similar Zr alloys^25^. In brief, the effect is caused by the changing defect density in the β-crystals. Upon heating in the single-phase field, the β-grains anneal thereby reducing the number of defects such as dislocations and forming perfect grains. As these grains reach a size comparable to the extinction length, which is accomplished in rather short time based on the high diffusivity of the β-phase, the kinematic condition breaks down and dynamic theory of diffraction dominates. Consequently, the diffraction intensities drop drastically. Recovery of the intensity is observed upon phase transformation when distortion and imperfections are induced by the formation of α. The observed increase in intensity for β-110 is explained by the reduced maximum temperature reached in every subsequent thermal cycle leading to slower phase transformations kinetics. This is also supported by the prolonged presence of β as visible in an increased intensity for HT2 compared to HT1 upon transformation to α′.

A detailed analysis of 2D diffractograms at selected time points throughout the thermal treatments is shown Fig. 4. The formed β is coarse grained based on the low amount of reflections observed (Fig. 4a). Consequently the obtained 1D diffractograms suffer from low grain statistics as it is evident from Fig. 2. Additionally, the broad β-110 reflection observed in Fig. 2 is indeed β after solidification, but due to its spread and low intensity is almost averaged out upon azimthal integration. The β-110 reflection at an angle of ~45° to the vertical is characteritic for solidification along <100> in the body-centered cubic crystal. The β-200 is observed in the high temperature region of HT2 as a single reflection (Fig. 4b). This diffraction spot has no diffuse scattering around it as it is passing through a thicker section of the specimen based on its downward diffraction direction with only the intense part being able to transmit and being detected. In that sense the specimen acts as a filter for the obtained signal, explaining the sharp reflection observed. Reflections in the upper-half of the detector clearly show thermally diffuse scattering evident as a halo around the diffraction spots. The α′ phase shows clustered blurred reflections on arc segments after melting and rapid solidification (Fig. 4c). Upon transformation into α′, the β-grain breaks down into several variants (sub-grains), hence the many more spots. Each spot is a small arc at sharp radius q – the width of the arc is the mosaic spread of that grain – on top of several subgrains. The groups of reflections are then formed by the mosaicity of the formed α′ with small misalignment between individual diffracting crystals^26^. After transforming to β and back-transformation upon cooling in HT1, numerous weak reflections disappear indicating grain coalescence in the formed α′ while the mosaic spread within the grains remains virtually unchanged (Fig. 4d) ^27^. This is associated with the increased intensity for the remaining strong reflections (compare to Fig. 3). Upon HT2 again weak reflections disappear, however in far smaller numbers (Fig. 4e,f). The observed temporary intensity increase of the β-110 reflection upon the β → α′ phase transformation is clearly visible by the formation of a sharp reflection (Fig. 4e, inset). The alignment of the observed β-110 and α′-0002 reflections fulfills the Burgers orientation relationship α(0001)||β{110}, α < 11$\overline{2}$0> ||β < 111>^28^. Additionally, the alignement of β-110 with α′-10 $\overline{1}$1 is observed which would be the condition for a Potter orientation condition^27^. With finishing the transformation to α′, the β-110 reflection vanishes until only α‘ is observed (Fig. 4f, inset). Based on these results it can be concluded that the phase orientation remains stable after initial rapid solidification and cooling. Upon following cycles less favourable orientations vanish, but most of the reflections in both β and α′ are observed repeatedly during thermal cycles.

### Lattice expansion upon rapid laser heating and cooling

Thermal expansion and phase transformations lead to changed lattice parameters of the crystal structure that are extracted from the peak positions upon analysis using the room temperature lattice parameter for α′ (Figure S3). The highest expansion of 1.4% is observed for the melting step where the α′-phase transforms to β and disappears within 200 ms. For HT1 and HT2 only a maximum expansion of 1% is obtained. In HT3 the β-transus is not reached and the expansion evolution demonstrates the comparably low heating rate of this final step. Detailed analysis shows a clear change in slope for lattice expansion in melting, HT1 and HT2. Comparing with the phase analysis in Fig. 2, this is caused by the starting α′ → β transformation. Despite weak diffraction signals impeding a clear identification of the β-phase, expansion in the α′-phase is very sensitive to the appearance of this second phase. The lattice expansion of the cubic β-phase is almost identical for the different thermal cycles. As β is not preserved to room temperature after cooling, expansion is calculated from the β-211 reflection using the published lattice parameter of 0.3246 nm for β in Ti-6Al-4V at room temperature^16^. Generally weak and partially intermittent diffraction intensities lead to scattered lattice expansion values. While melting and HT2 completely overlap, slightly larger expansion is observed after HT1. Assuming the β-phase lattice expansion is caused only by thermal expansion, the martensite start temperature is estimated around 923 K by comparison with published lattice parameters at elevated temperature^16^. This estimated value is at the lower boundary of literature values for Ti-6Al-4V^22^ which is explained by the high cooling rate obtained in laser-based heating and rapid cooling.

### Surface temperature distribution and evolution

**Figure 5:**
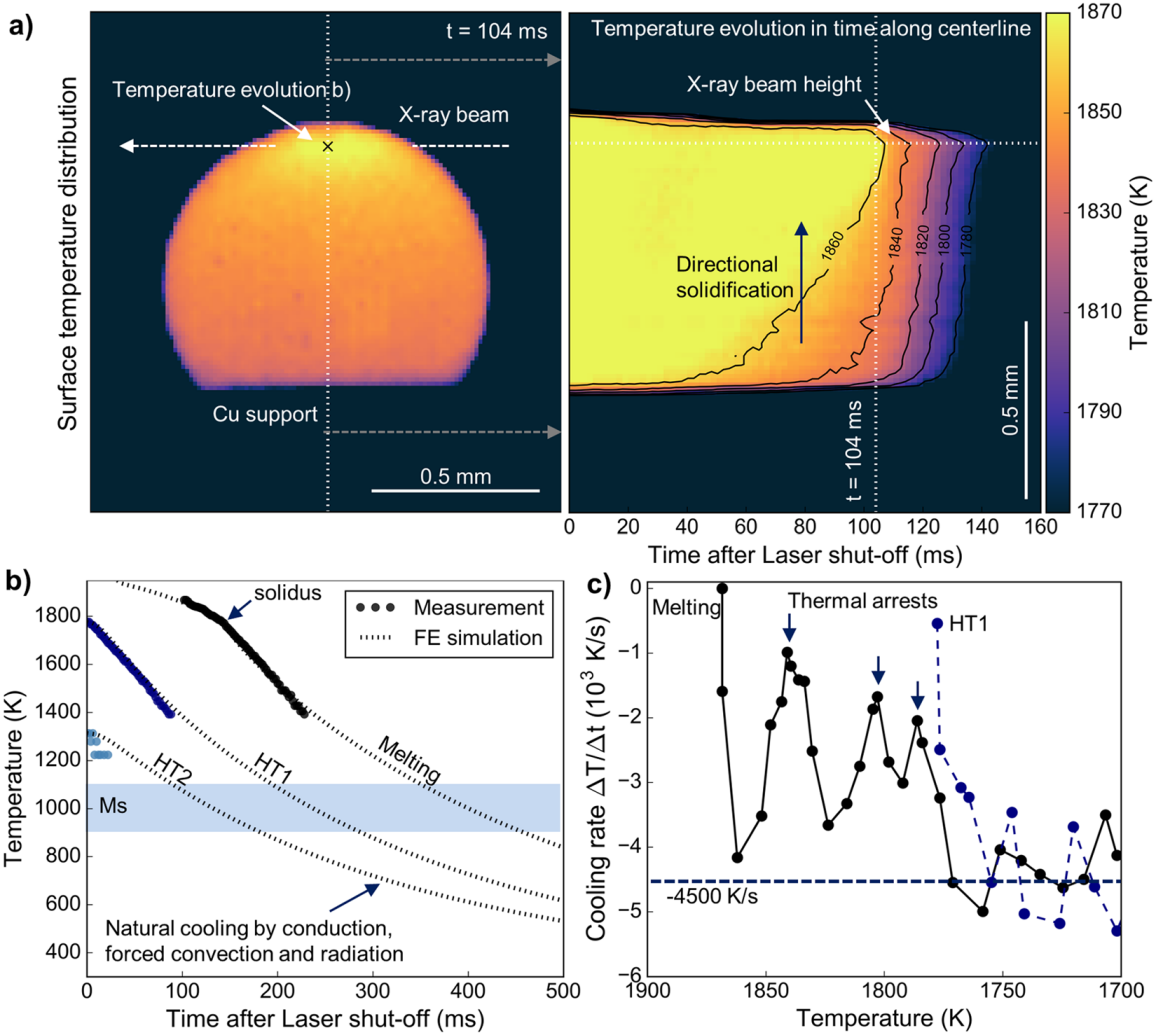
Surface temperature distribution and evolution calculated from off-axis high-speed imaging data After initial melting, the solidification front proceeds from bottom to top against the temperature gradient imposed by the copper support. A cross-section along the centre line shows an incubation period of 40 ms before the solidification front proceeds through the specimen within 64 ms (**a**). The temperature evolution at the position of the X-ray beam is extracted for the first three cycles. The typical range of reported martensite start temperatures (Ms) is indicated (**b**). The cooling rate shows three distinct thermal arrests indicating solidification events upon cooling after the melting cycle. The obtained cooling rate after both melting and HT1 is -4500 K/s (**c**).

The surface temperature distribution and evolution is calculated from the high-speed imaging data using a radiometric model and prior camera calibration. This data is used to estimate solidification front velocities, thermal gradients and cooling rates. These parameters allow comparing the obtained results with real world processing results. Figure 5a reveals a strong vertical and reduced horizontal thermal gradient. Unidirectional heat extraction into the Cu support quickly cools the specimen after the lasers are shut-off. The X-ray beam traverses the specimen at an inclined angle to the figure normal as imaging is performed 45° off-axis. The evolution of the thermal gradient along the centreline of the specimen with respect to the camera position reveals the vertical progression of solidification from bottom to top. Based on the changing isotherms the vertical solidification speed is estimated at 10.3 mm/s. This value is in the same range as typical processing speeds in direct metal deposition of Ti-6Al-4V^29^. Figure 5b shows extracted surface temperature data at the X-ray beam height. The camera exposure time is adjusted to cover the solidification and initial cooling with the limited dynamic range of the CCD sensor. Consequently, the high temperature liquid phase after laser-shut-off is not covered. The low temperature branch is not measured as the radiation of visible light becomes insufficient for this exposure parameters below 1400 K. Supporting finite element modelling is performed to obtain estimated cooling curves beyond the measured range. Upon solidification, a change in slope is observed indicating full solidification at a surface temperature of 1777 K (note that the specimen surface cools faster than the interior and thus the solidus temperature is under-estimated). The maximum temperature after HT1 is 1778 K and clearly lower than the equilibrium solidus of Ti-6Al-4V at 1878 K^22^. This agrees with the X-ray diffraction results where the β-phase exists throughout HT1 (Fig. 2). In HT2 the maximum observed temperature is 1314 K, slightly higher than the β-transus of this alloy at 1267 K. It must be noted that at this temperature the CCD reaches the lower limit of its dynamic range and the measured temperature is subject to larger errors. Based on the reported range of martensite start temperatures in Ti-6Al-4V and the estimation using the β lattice strain the time to transformation can be estimated from the calculated cooling curves from finite element modelling. For melting the β → α′ transformation is expected after around 450 ms, for HT1 after around 250 ms and for HT2 after about 200 ms. These results are in good agreement with the obtained results from X-ray diffraction (Figs 2 and 3).

Figure 5c reveals a solid-state cooling rate of 4500 K/s after both melting and HT1. The cooling rate is calculated as the piece-wise thermal gradient of the measured temperature signal. The obtained cooling rate is close to reported 4800 K/s for direct metal deposited Ti-6Al-4V^30^. Detailed analysis of the cooling rate after melting reveals three distinct thermal arrests where the cooling rate is strongly decreased. These recalescence events are attributed to solidification of the β-phase from the undercooled liquid. This is also evident from the rapid formation and following intermittent presence of the β-211 reflection in Fig. 2. Assuming that the surface temperature of the first recalescence event is close to the real bulk temperature in the beam path, the undercooling of the melt upon formation of β is estimated at 37 K. Such a comparably low value is explained by the release of the enthalpy of fusion from the solidification front preventing a deep undercooling in the remaining liquid. Additionally, nucleation is not a limiting factor as β can readily grow from the substrate formed by already solidified Ti-6Al-4V.

## Conclusion

In summary, it has been shown that

- a setup has been developed that allows following rapid heating and solidification with specific emphasis on high spatial and temporal resolution to clearly separate the contributions from liquid and solid phases;
- cyclic laser-based heating and subsequent cooling can be performed in rapid succession using the developed approach;
- Ti-6A-4V repeatedly undergoes the β ↔ α′ phase transformation upon rapid heating and cooling. At room temperature only α′ is observed. 2D diffractograms reveal reduced mosaicity upon continued thermal cycling and alignment of α′ and β according to the Burgers and Potter orientation relations.
- the lattice expansion in the α′-phase are sensitive to the α′ → β phase transformation upon rapid heating. During cooling lattice expansion shows an identical evolution. Additionally, the c/a ratio of the hexagonal phase is increased after melting and relaxes upon following thermal cycles. The martensite start temperature is estimated at 923 K from the lattice strain of the β-phase.
- the solidification front velocity and the cooling rate are estimated from high-speed imaging data at 10.3 mm/s and 4500 K/s, respectively. These values are in agreement with reported parameters for direct metal deposition of Ti-6Al-4V.

The advantages of the presented setup are (i) a window-less operation minimizing absorption losses and maximizing temporal resolution; (ii) a high spatial and temporal resolution based on an intense micro-focused X-ray beam and a fast single-photon counting detector; (iii) a clear separation of the contributions from liquid and solid phases due to a low thermal gradient along the X-ray beam path in the specimen and (iv) the combination of X-ray diffraction with surface temperature information from *in situ* high-speed imaging to obtain solidification front velocities, cooling rates and undercooling temperatures. These values are crucial to compare the obtained results with real processing. A limitation of the presented setup is the counting statistics as only a limited number of grains is in the beam path that fulfils the diffraction conditions. Sacrifices are also made for the peak (angular) resolution due to a reduced sample-detector distance and the use of a 2D detector to achieve the selectivity and temporal resolution of the presented setup.

## Methods

### Materials

In this work, commercial Ti-6Al-4V (ASTM Grade 5, Bibus Metals, Switzerland) is studied. The used alloy batch contains 6.30% Al and 4.15% V. The main impurities are 0.17% O_2_, 0.15% Fe, 0.02% C, 0.01% N_2_ and 0.005% H_2_ (all wt.%). The measured β-transus is 1267 K. Specimens for *in situ* diffraction experiments are prepared by arc melting small amounts (2 mg) in a water-cooled Cu crucible under Ar atmosphere. Upon rapid solidification, a near-spherical specimen with a typical diameter of 1 mm is formed by surface tension (Figs 1a and 5a).

### Rapid laser melting and cooling setup

The specimen is supported on a tapered tip machined from Cu to maximize heat extraction (Fig. 1). Heating is performed for 1.5 s by a laser heating system^31^ consisting of two diode lasers (Apollo Instruments, USA) operating at 980 nm with a maximum power output of 150 W each and a rectangular beam profile with (0.2 × 1) mm^2^. Both lasers are inclined by 20° to the horizontal aiming at water-cooled beam dumps to avoid damages in case no specimen is in the beam path. The complete laser rig has motorized axes in 3 dimensions. Shielding gas (Ar 4.8) is supplied by a laminar flow nozzle (Arc-Zone, USA) in a radius of 15 mm around the specimen. The resulting vertical Ar column provides the necessary inert atmosphere enabling a window-less setup minimizing absorption. Cyclic heating and cooling is performed on a single specimen in direct succession with a total measurement time of 10 s per cycle.

### *In situ* high-speed micro X-ray diffraction

All experiments are performed at the undulator-based microXAS beamline of the Swiss Light Source (SLS) located at the Paul Scherrer Institut, Switzerland. A micro-focused monochromatic X-ray beam with a FWHM of (9 × 30 μm^2^) and 17.3 keV is provided by a Si(111) monochromator and Kirkpatrick-Baez (KB) mirror optics. Diffracted photons are recorded by an EIGER × 1 M single photon counting detector (Dectris, Switzerland) located down-stream of the specimen (Fig. 1). The total active area is 1030 × 1065 pixels at 75 μm pixel size. The detector is operated at a continuous frame rate of 1 kHz with 0.99 ms exposure time for individual frames. The direct beam is aligned to the module border and shielded by a beam stop. α-Al_2_O_3_ is measured as a calibration standard.

### Off-axis high-speed imaging

High-speed imaging is performed using a Motion Xtra HG-100 K high-speed camera (Redlake, USA) operated at an acquisition rate of 500 Hz and 200 μs dwell time. Imaging is performed 45° off-axis with respect to the X-ray beam. The temperature is calculated from the measured intensity on the CCD sensor according to ref.^23^

$${I}_{CCD}=\frac{a\cdot {c}_{1L}}{\exp (\frac{{c}_{2}}{b\cdot T})}$$

where *I*_*CCD*_ is the intensity on the CCD chip, ${c}_{1L}=2h{c}^{2}$ is the first radiation constant with $h$ being the Planck constant and $c$ being the speed of light in vacuum, ${c}_{2}=hc/k$ is the second radiation constant with *k* being the Boltzmann constant, *T* is the temperature and *a* = 7.95·10^22^ W^−1^·m^−2^ and *b* = 7.32·10^−7^ m are the camera-dependent parameters.

### Data analysis

2D diffraction diffractograms recorded by the EIGER × 1 M are screened at the beam line for quality control using the ALBULA viewer (Dectris, Switzerland). Detector distance calibration, background subtraction, azimuthal integration and averaging are performed using XRDUA v7.1.3.1^32^. The background is determined from a series of 1000 averaged 2D diffractograms obtained without a specimen. All diffraction data is shown with the momentum transfer $q=4\cdot \pi \cdot \,\sin \,\theta /\lambda$, where 2*θ* is the diffraction angle and *λ*is the X-ray wavelength. Integrated peak intensities, peak positions and lattice strains are obtained from least-square fitting Gaussian functions to the 1D diffractograms at every point in time. The lattice strains are calculated from the planar distance $d=2\cdot \pi /q$. Room temperature distances are used for hexagonal α′, literature values for body centered cubic β^16^. The analysis and plotting of X-ray diffraction and imaging is performed using MATLAB (MathWorks, USA) and Python (Anaconda distribution, Continuum analytics, USA) in combination with the IPython^33^, matplotlib^34^, SciPy and NumPy^35^ packages. Perceptually uniform colour maps are retrieved from the cmocean^36^ package.

### Finite element modelling

Supporting finite element transient heat transfer modelling is performed in Abaqus/CAE 6.14-2 (3DS Simulia) with a 160,000-element hexahedral (DC3D8) mesh. The Cu support is modelled only in the vicinity of the specimen to reduce computational cost. The model includes conductive heat transfer between the specimen and the Cu support as well as surface radiation from the specimen to the ambient surrounding and forced convective cooling by the Ar shielding gas. Initial temperatures are taken from high-speed camera imaging thermal data. Published thermophysical data is used for Ti-6Al-4V^37–39^, Cu^40^ and Ar^41^.

## Electronic supplementary material

Supplementary Information
