# Supplementary material for: In situ investigation of phase transformations in Ti-6Al-4V under additive manufacturing conditions combining laser melting and high-speed micro-X-ray diffraction
Source: Sci Rep. 2017 Nov 27;7:16358. doi: 10.1038/s41598-017-16760-0 (PMC5703903; doi:10.1038/s41598-017-16760-0)
Supplement: Supplementary file 1 — Supplementary Information [file 41598_2017_16760_MOESM1_ESM.pdf]

In situ investigation of phase transformations in Ti-6Al-4V under additive manufacturing conditions combining laser melting and high-speed micro-X-ray diffraction

C. Kenel[1,2], D. Grolimund[3], X. Li[2], E. Panepucci[3], V.A. Samson[3], D. Ferreira Sanchez[3], F. Marone[3], C. Leinenbach[2,*]

[1] Northwestern University - Department of Materials Science and Engineering, 2200 Campus Drive, Evanston, IL 60208, USA

[2] Empa - Swiss Federal Laboratories for Materials Science and Technology, Überlandstrasse 129, 8600 Dübendorf, Switzerland

[3] Paul Scherrer Institut, Swiss Light Source, 5232 Villigen PSI, Switzerland

*) corresponding author: christian.leinenbach@empa.ch, Tel: +41 58 765 4518

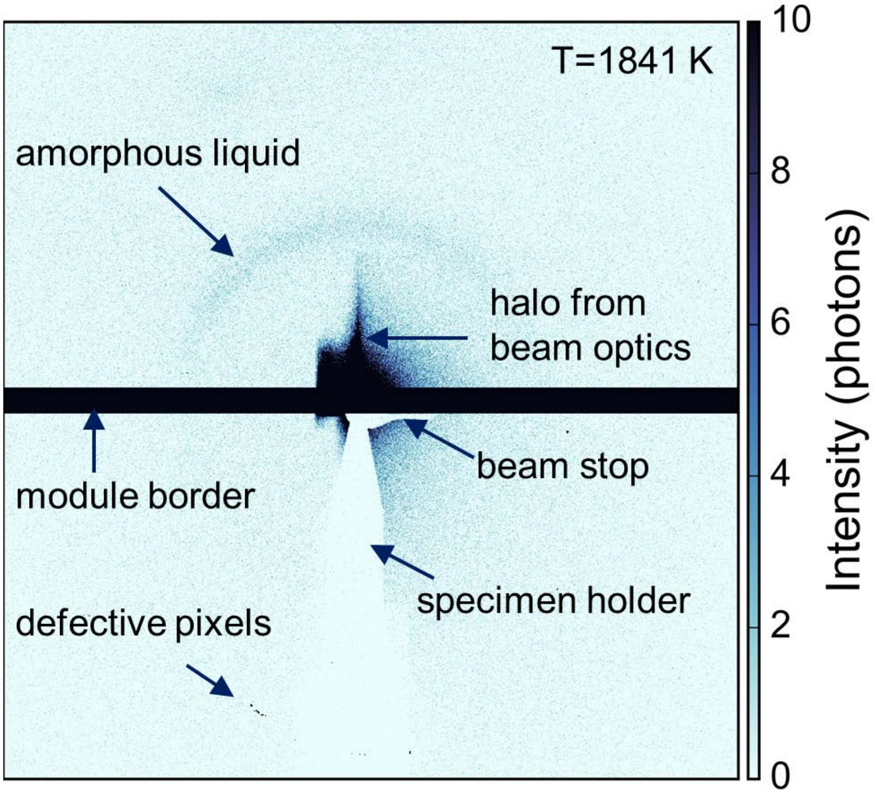

Supplementary Figure S1: Scattered signals from amorphous and crystalline phases combined with features from beam optics, the specimen holder, the beam stop, defective pixels and the module border

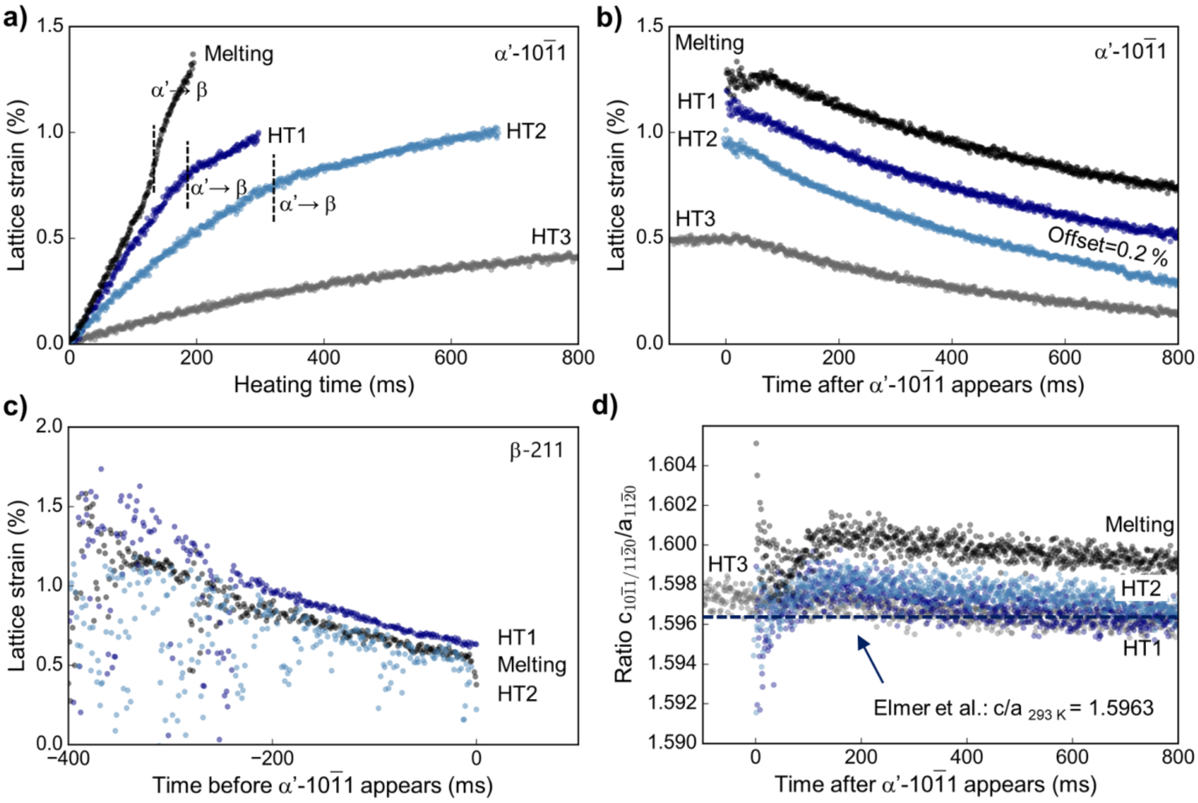

Supplementary Figure S2: Lattice strain evolution upon rapid heating and cooling The lattice strain is obtained from fitting the 1D spectra at every point in time. The strain along α′(10$\bar{1}$1) reveals thermal expansion and the onset of phase transformations (¦) upon rapid heating with decreasing laser power (a). For the corresponding cooling cycles the measured strain perfectly overlaps for melting, HT1 and HT2. These curves are shown with an offset of 0.2 % (b). Weak diffraction signals from the β phase reveal increased lattice strain with decreasing laser power prior to β → α′ transformation (c). The c/a ratio calculated from the α′(10$\bar{1}$1) and α′(11$\bar{2}$0) peaks of the hexagonal α′ phase reveals c/a anisotropy after melting and values close to literature values after HT1 to HT3 indication relaxation upon *in situ* heat treatment (d).

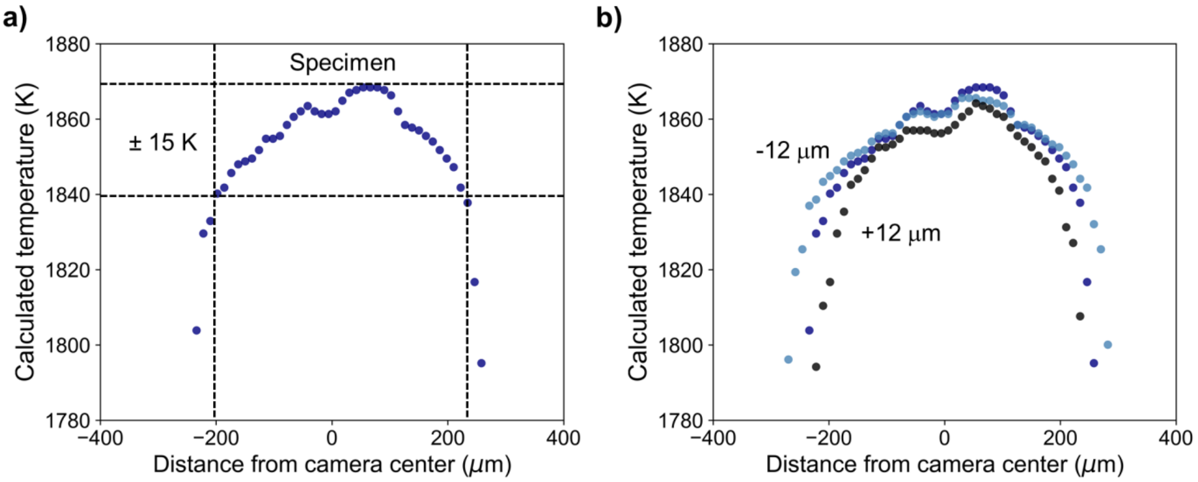

Supplementary Figure S3: a) Horizontal temperature calculated from the CCD camera signal; b) variation of horizontal temperature for vertical shifts of the sample of +12 µm and -12 µm; The maximum possible vertical movement of the specimen can be calculated from its thermal expansion. Assuming heating of the complete specimen to the melting temperature and taking the data from Elmer et al. for α <1273 K and β >1273 K, the calculated maximum vertical expansion is 35 micrometers from solidification to room temperature.
